# LncRNAs are regulated by chromatin states and affect the skeletal muscle cell differentiation

**DOI:** 10.1111/cpr.12879

**Published:** 2020-08-07

**Authors:** Xiaolong Qi, Mingyang Hu, Yue Xiang, Daoyuan Wang, Yueyuan Xu, Ye Hou, Huanhuan Zhou, Yu Luan, Zhangxu Wang, Weiya Zhang, Xinyun Li, Shuhong Zhao, Yunxia Zhao

**Affiliations:** ^1^ Key Lab of Agricultural Animal Genetics, Breeding, and Reproduction of Ministry of Education Huazhong Agricultural University Wuhan China; ^2^ The Cooperative Innovation Center for Sustainable Pig Production Wuhan China

**Keywords:** C2C12, chromatin state, differentiation capacity, lncRNA, muscle satellite cell, transcription factor

## Abstract

**Objective:**

This study aims to clarify the mechanisms underlying transcriptional regulation and regulatory roles of lncRNAs in skeletal muscle cell differentiation.

**Methods:**

We analysed the expression patterns of lncRNAs via time‐course RNA‐seq. Then, we further combined the ATAC‐seq and ChIP‐seq to investigate the governing mechanisms of transcriptional regulation of differentially expressed (DE) lncRNAs. Weighted correlation network analysis and GO analysis were conducted to identify the transcription factor (TF)‐lncRNA pairs related to skeletal muscle cell differentiation.

**Results:**

We identified 385 DE lncRNAs during C2C12 differentiation, the transcription of which is determined by chromatin states around their transcriptional start sites. The TF‐lncRNA correlation network showed substantially concordant changes in DE lncRNAs between C2C12 differentiation and satellite cell rapid growth stages. Moreover, the up‐regulated lncRNAs showed a significant decrease following the differentiation capacity of satellite cells, which gradually declines during skeletal muscle development. Notably, inhibition of the lncRNA *Atcayos* and *Trp53cor1* led to the delayed differentiation of satellite cells. Those lncRNAs were significantly up‐regulated during the rapid growth stage of satellite cells (4‐6 weeks) and down‐regulated with reduced differentiation capacity (8‐12 weeks). It confirms that these lncRNAs are positively associated with myogenic differentiation of satellite cells during skeletal muscle development.

**Conclusions:**

This study extends the understanding of mechanisms governing transcriptional regulation of lncRNAs and provides a foundation for exploring their functions in skeletal muscle cell differentiation.

## Graphical abstract

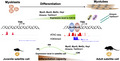

## 1 INTRODUCTION

The formation of myotubes is a necessary step in the complex and multi‐stage process of muscle development, which is affected by many cytokines and self‐proteins.^1^ The vitality of satellite cells is essential for maintaining the stability of skeletal muscle tissue.^2^ Moreover, both the capacity for differentiation and the number of satellite cells decrease with age during skeletal muscle development in mice.^3^ However, the current understanding of gene regulation during the processes of skeletal muscle cell differentiation and functional decline in satellite cells remains incomplete, especially regarding the role of long non‐coding RNAs (lncRNAs).

Transcription factors (TFs), such as the myogenic regulatory factors MyoD family, the MEF2 family (MEF2A‐D) and others, have been shown to determine the characteristics of skeletal muscle cells.^4^, ^5^, ^6^ The MyoD, MyoG and Mef2c are specifically involved in the regulation of myogenic differentiation, and their expression is significantly reduced and delayed in satellite cell senescence.^7^, ^8^ Although *Heyl* and *Hey1* are considered potential effectors of the Notch pathway to inhibit myogenic differentiation, only constitutive expression of *Hey1* blocked myogenesis.^9^ Another study found that the weight and size of *Heyl/1* double‐knockout mice decreased, which indicates that *Heyl* has a positive effect on muscle development.^10^ These previous findings demonstrate that the expression of TFs can be used as a molecular marker of cell differentiation state and function.

A growing body of evidence supports the finding of a close relationship between lncRNA function and skeletal myogenesis, and muscle diseases.^11^, ^12^ The *Linc‐MD1* RNA was reported to be involved in skeletal muscle differentiation through regulation of myogenic TFs.^13^, ^14^ Similarly, *Linc‐YY1* promotes myogenic differentiation via the interactions with YY1 and regulates satellite cell activation/proliferation by regulating *Pax7* expression.^6^ *LncMyoD* has been found to induce myogenic differentiation through disrupting the cell cycle.^15^ The lncRNA *Trp53cor1*, also known as *LincRNA‐p21*, has been shown to participate in repressing cell proliferation and smooth muscle cell apoptosis.^16^, ^17^ Collectively, these discoveries provide strong evidence of a contribution by lncRNAs to skeletal muscle development, although their transcriptional regulation and regulatory roles have not been well‐studied at the whole genome level.

In this work, we systematically analysed expression patterns of lncRNAs using RNA‐seq over a time course spanning from proliferation to differentiation of C2C12 myoblasts and mouse satellite cells across a range of ages to understand their regulatory functions. Moreover, the transcriptional regulation of lncRNAs was examined by combining ChIP‐seq and ATAC‐seq data. Our study provides new insights into the transcriptional regulation of lncRNAs and the mechanisms underlying their regulatory roles in skeletal muscle cell differentiation.

## 2 MATERIALS AND METHODS

### 2.1 Sample collection and cell culture

The C2C12 mouse myogenic cell line was acquired from the Cell Bank of Wuhan University and cultured in 1 × DMEM basic (Gibco‐BRL) with 20% foetal bovine serum (Gibco 10099133) in 5% CO_2_ at 37°C. Myogenic differentiation was initiated upon reaching 90% confluence by switching the cells to DMEM containing 3% horse serum (Hyclone SH30074.03) and then cultured for 12, 24, 48, 60 and 96 hours. Skeletal muscle satellite cells were isolated from the hindlimb muscle of C57BL/6 mice at weeks 2, 4, 6, 8, 10 and 12, which were represented as W2, W4, W6, W8, W10 and W12, respectively. The detailed isolation process for satellite cells is described in our previous report.^3^

### 2.2 Analysis of differentially expressed lncRNAs

The mouse reference genome and GTF files of lncRNAs and protein‐coding genes were downloaded from the GENCODE database version M11 (https://www.gencodegenes.org/mouse/). The expression levels of protein‐coding genes and lncRNAs were quantified by tophat v2.1.1^18^ and the htseq‐count script of htseq v0.6.0.^19^ Differential expression analysis of C2C12 was performed on transcriptomic data collected between proliferation (cells cultured in growth medium, GM) and differentiation (cells cultured in differentiation medium, DM 12, 24, 48, 60 and 96 hours) by edger.^20^ Differentially expressed (DE) genes were identified based on a fold change threshold value of (|log_2_FC| ≥ 1) and *P* value (*P* < .05).

### 2.3 ATAC‐seq experiments and data quality control

ATAC‐seq was conducted primarily following previous studies.^21^, ^22^ In our study, about 50 000 C2C12 in the proliferation and differentiation (60 hours) were lysed in 50 μL ATAC‐seq lysis buffer (10 mmol/L Tris‐HCl, pH 7.4, 10 mmol/L NaCl, 3 mmol/L MgCl_2_, 0.1% NP40, 0.1% Tween‐20 and 0.01% digitonin) and incubated on ice for 5 minutes. Add 1 mL cold ATAC‐buffer (10 mmol/L Tris‐HCl, pH 7.4, 10 mmol/L NaCl, 3 mmol/L MgCl_2_ and 0.1% Tween‐20) was added to stop the lysis. The cells were further centrifuged at 500 RCF for 10 minutes at 4°C to collect the cell pellet after removing the supernatant. The following transposition reaction mix (25 µL Illumina Tagment DNA buffer Cat#: 15027866, 16.5 µL 1 × PBS, 0.5 µL 1% digitonin, 0.5 μL 10% Tween‐20, 2.5 μL ddH_2_O and 5 μL Illumina Tagment DNA enzyme (Cat#: 15027916) was added to the cell pellet and incubated at 37°C for 1 hour on thermocycler. ZYMO RESEARCH DNA Clean & Concentrator‐5 Kit (Cat#: D4013) was used to purify the transposed DNA. Library preparation was following the instruction of previous study.^21^ Final library was electrophoresed in a 2% high‐resolution agarose gel and 100‐1000 bp fragments were cut out and sequenced by using Illumina HiSeq X Ten PE150 platform. The information of ATAC‐seq data is shown in Table S1 and Figure S1.

### 2.4 ATAC‐seq and ChIP‐seq data processing

Transcription factor (MyoD, MyoG, Cebpb, Usf1 and Max^23^) and histone modification (H3K4me3^23^ and H3K4me1^24^) ChIP‐seq data were downloaded from the previous studies. These data were obtained from C2C12 cells at proliferation and differentiation stages.

The ATAC‐seq reads were mapped to the mouse M11 reference genome from GENCODE database using bowtie2 v2.3.4.1.^25^ Peaks for each replicate were called individually with macs2 v2.1.0^26^ and merged with bedtools v2.26.0.^27^ After merging, peak positions were defined as open chromatin regions. deeptools v2.5.2^28^ was used for statistical analysis of the ATAC‐seq peak signals. The Normalize.quantiles function of preprocesscore package v1.40.0^29^ in r v3.5.1 was used to normalize the ATAC‐seq signal. ChIP‐seq data were subjected to a similar process as ATAC‐seq reads.

### 2.5 Integrated analysis of DE lncRNAs, ATAC‐seq and ChIP‐seq

Differentially expressed (DE) lncRNAs with a counts per million (CPM) less than 1 in both GM and DM60 hours time points were filtered. The DE lncRNAs were classified as up‐ (log_2_FC > 0) or down‐regulated (log_2_FC < 0) depending on the log_2_FC in expression for each lncRNA between GM and DM60 hours time points in C2C12 cells. The same number of lncRNAs was randomly selected from non‐DE lncRNAs as a control. The lncRNAs with transcription start sites (TSS‐, upstream 2.5 kb and downstream 1.5 kb) that overlapped with ATAC peaks were identified as open chromatin‐associated DE lncRNAs using bedtools v2.26.0.^27^ The log_2_FC of ATAC‐seq signals between GM and DM60 hours around the TSS of the above DE lncRNAs and non‐DE lncRNAs was calculated based on ATAC‐seq signals described in Section 2.4.

Histone ChIP‐seq and ATAC‐seq usually have corresponding but not precisely co‐localized peaks around the TSS of lncRNAs. In this step, the upstream and downstream regions flanking ATAC‐seq peaks were both extended 1 kb and the peaks were again merged using bedtools with ‘‐d 0’ to obtain an accurate ChIP‐seq and ATAC‐seq co‐localized peak region and histone modification signal. Then, the DE lncRNAs associated with open chromatin were divided into three categories: lncRNAs associated with ATAC and H3K4me1, lncRNAs associated with ATAC and H3K4me3, and lncRNAs associated only with ATAC.

Subsequently, the ChIP‐seq signal for H3K4me3/H3K4me1 and the ATAC‐seq signal in the extended peak regions were calculated using deeptools v2.5.1 and normalized with the Normalize.quantiles function of preprocesscore package v1.40.0^29^ in r v3.5.1. In each category, the log_2_FC of the ATAC‐seq or ChIP‐seq signals located within 2 kb of ATAC‐seq peaks of DE lncRNA TSS was calculated using the same method as log_2_FC expression analysis.

The motif analysis in this study was performed using the findMotifs.pl function of homer software.^30^

### 2.6 Correlation analysis

Weighted correlation network analysis (WGCNA)^31^ was used to identify patterns of co‐expression between significant DE genes and lncRNAs of C2C12 cell line RNA‐seq data. The WGCNA package in the r v3.5.1 environment was applied to develop a weighted correlation network based on the normalized count matrix produced during differential expression analysis of lncRNAs using edger.^20^ The Pearson correlation analysis was also used to identify significantly correlated DE lncRNA‐gene pairs in C2C12 (*P* < .05). DAVID Bioinformatics Resources 6.8 (https://david.ncifcrf.gov/summary.jsp) was used for GO term functional annotation of significantly enriched genes.

### 2.7 Gene set enrichment analysis (GSEA)

gsea ^32^ software v3.0 was used to interpret the gene expression data derived from satellite cell RNA‐seq data. Phenotype labels included W4 vs W2, W6 vs W2, W8 vs W2, W10 vs W2, and W12 vs W2. The DE lncRNAs identified in C2C12 cells that were correlated with *MyoD*, *MyoG*, *Mef2c* and *Heyl* were divided into up‐regulated and down‐regulated lncRNA groups based on changes in their expression during different stages of differentiation in C2C12 cells. Gene sets were mapped to the pre‐ranked gene list to calculate the enrichment score.

### 2.8 qPCR

Total RNA from C2C12 and satellite cells was extracted with TRIzol reagent (Invitrogen 15596026) according to the manufacturer's instructions. cDNA was obtained via reverse transcription of 1 μg of RNA using a High‐Capacity cDNA Reverse Transcription kit (Thermo Fisher 4374967). THUNDERBIRD SYBR qPCR Mix (Toyobo) was used with a CFX384 real‐time PCR system (Bio‐Rad). All primer sequences in this study are listed in Table S2.

### 2.9 Cell transfection

For RNAi assays, siRNA constructs targeting *Atcayos* and *Trp53cor1* were transfected into satellite cells using Lipofectamine 2000 (Invitrogen) according to the manufacturer's instructions. The siRNA and negative control were provided by RiboBio lncRNA Smart Silencer (RiboBio). The target sequences in this study were listed in Table S3.

### 2.10 Immunofluorescence of satellite cells

Cultured cells were washed with 1 × PBS and fixed in 4% paraformaldehyde for 10 minutes at room temperature. After 10 minutes of penetration in ice‐cold 0.3% Triton X‐100, cells were incubated in blocking buffer (3% BSA, 0.25% Triton X‐100 and 10% FBS in PBS) for 1 hour to block non‐specific binding. Then, cells were incubated with anti‐myosin primary antibody (Monoclonal Anti‐Myosin, Sigma M4726, 1:200) at 4°C overnight with gentle shaking. Cells were further washed four times with 1 × PBS and then incubated with anti‐mouse IgG (H+L), F (ab’) 2 Fragment (Alexa Fluor™ 555 Conjugate; CST, 4409). A Nikon Eclipse TE2000‐S microscope (Nikon) was used to observe the fluorescence.

### 2.11 Western blot

Cells were lysed in RIPA buffer (Sigma R0278) with phosphatase and protease inhibitors on ice for 30 minutes. Total protein was electrophoresed in 10% SDS‐PAGE gel and transferred onto a PVDF membrane (Millipore). The membranes were blocked in 5% skim milk for 2 hours at room temperature and then incubated overnight at 4°C with primary antibody against Myosin (Sigma M4276, 1:800), MyoG (Abcam ab1835, 1:500) or β‐tubulin (Sungene KM9003, 1:1000). After washing four times in TBST buffer (0.1% Tween‐20 in TBS), the PVDF membranes were incubated with horseradish peroxidase (HRP)‐conjugated secondary antibodies (Beyotime A0216) for 1 hour at room temperature. The PVDF membranes were then washed five times with TBST and treated with Immobilon Western Chemiluminescent HRP Substrate. HRP signals were captured with an ImageQuant LAS4000 mini system (GE Healthcare Bio‐Sciences).

## 3 RESULTS

### 3.1 Dynamic expression of lncRNAs during C2C12 myoblast differentiation

**FIGURE 1:**
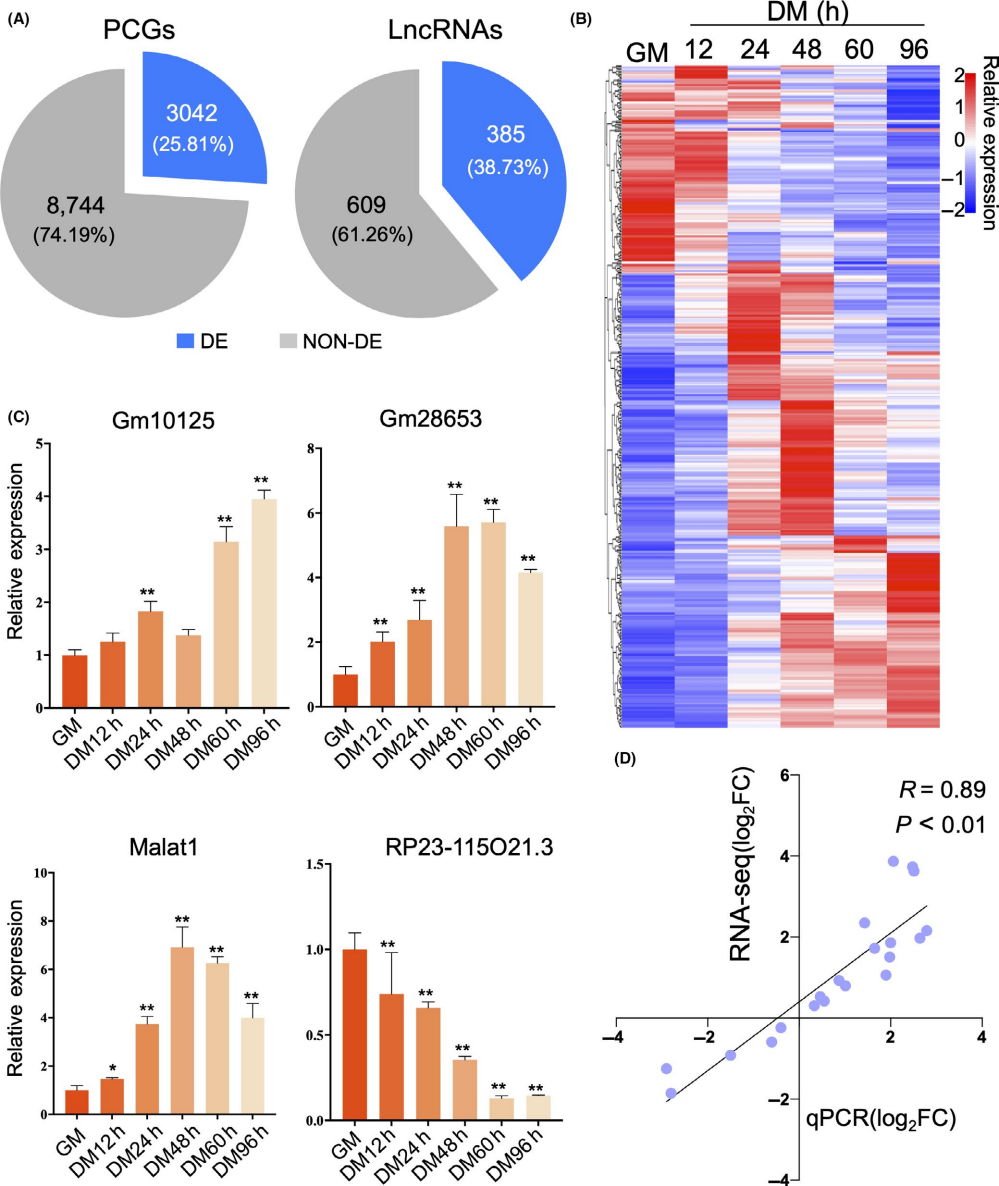
Identification of differentially expressed (DE) lncRNAs during C2C12 myoblast differentiation. A, Statistical results of DE protein‐coding genes (PCGs) and lncRNAs. B, Heat map of expression profiles of DE lncRNAs from the proliferation stage (GM) to the differentiation stage at 96 h (DM) (n = 3, |log_2_FC| > 1, *P* < .01). C, qPCR validation of RNA‐seq differential expression analysis using four randomly selected DE lncRNAs in differentiating C2C12 myoblasts (n = 3). *Tubulin* was used as the internal control. ‘*’ and ‘**’ indicate *P* < .05 and *P* < .01, respectively. Fold change in relative expression was based on expression levels during the proliferation stage. D, Scatter plot of the log_2_FC correlation between RNA‐seq (*y*‐axis) and qPCR (*x*‐axis)

LncRNAs are involved in multiple biological processes in skeletal muscles.^11^ Through the GENCODE project initiative, 9989 lncRNAs have been identified in the mouse genome. Among them, expression of 994 lncRNAs was detected in C2C12 proliferation and differentiation cells via the rRNA depletion strand‐specific RNA‐seq method used in our study. Here, 385 (38%) of DE lncRNAs and 3042 (25%) of DE protein‐coding genes (PCGs) were identified via RNA‐seq over a time course spanning the C2C12 myoblast differentiation process (Figure 1A). Visualization by heat map revealed dynamic changes in the expression of these DE lncRNAs during C2C12 myoblast differentiation (Figure 1B). To verify the accuracy of our expression pattern analysis, we randomly selected four DE lncRNAs for qPCR validation, namely *Gm10125*, *GM28653* and *Malat1*, which were up‐regulated, and *RP23‐115O21.3*, which was down‐regulated (Figure 1C). We found a high correlation between RNA‐seq and qPCR (*R* = .89, *P* < .01; Figure 1D), confirming the reliability of our differential expression analysis.

### 3.2 Chromatin states control lncRNA expression during C2C12 myoblast differentiation

**FIGURE 2:**
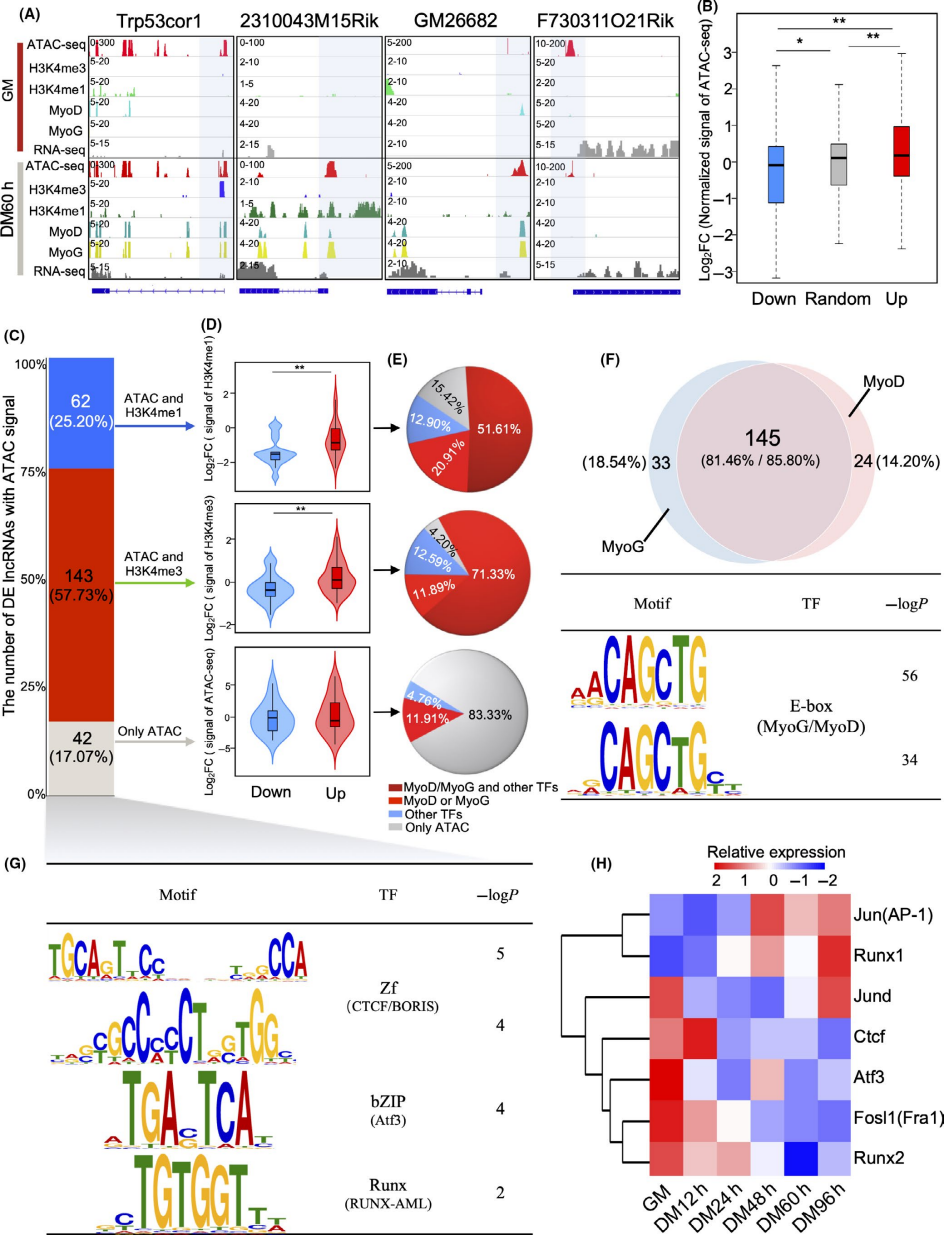
Differential expression of lncRNAs altered by changes in chromatin state during C2C12 myoblast differentiation. A, Changes in chromatin state around the transcription start site of DE lncRNAs between proliferation (GM) and 60 h of differentiation (DM60 h). B, Box plot showing the changes in ATAC‐seq intensity around the transcriptional start site (TSS) of open chromatin‐associated DE lncRNAs between GM and DM60 h in C2C12 myoblasts. Blue, red and grey boxes represent the log_2_FC of open chromatin around the TSS of down‐ and up‐regulated open chromatin‐associated DE lncRNAs and randomly selected non‐DE lncRNAs, respectively. ‘*’ and ‘**’ indicate *P* < .05 and *P* < .01, respectively. C, Bar plot showing the DE lncRNAs associated with open chromatin. The lncRNAs were classified by the type of histone modifications identified by ChIP‐seq in the open chromatin regions around their TSS. D, Changes in ChIP‐seq/ATAC‐seq intensity for each category of DE lncRNAs associated with open chromatin between C2C12 myoblast GM and DM60 h stages. E, TF binding states around the TSS regions for each category of lncRNA associated with open chromatin in (C). F, Venn diagram and motif enrichment showing the co‐localization of MyoD and MyoG binding sites. Motif enrichment analysis was performed using the open chromatin regions around DE lncRNAs. G, Motif enrichment of the open chromatin regions of DE lncRNAs associated only with the ATAC‐seq signal in (C). H, Heat map showing the relative expression profile of associated TFs from (G)

Following identification of DE lncRNAs and analysis of their differential expression, we conducted ATAC‐seq to observe changes in open chromatin states in regions adjacent to the TSS of the above DE lncRNAs (Figure 2A). We found that 247 (63.90%) DE lncRNAs, with CPM (counts per million) ≥1 in either GM or DM60 hours, had associated ATAC‐seq peaks around their TSSs (2.5 kb upstream, 1.5 kb downstream), and were therefore designated as open chromatin‐associated DE lncRNAs. Moreover, the log_2_FC in DM60 hours vs GM comparison of the open chromatin ATAC‐seq signal for up‐regulated DE lncRNAs was significantly higher than those of randomly selected non‐DE lncRNAs (*P* < .05) and down‐regulated DE lncRNAs (*P* < .01; Figure 2B). Thus, these results suggested that the change in open chromatin states was the primary reason for the differential expression of lncRNAs during C2C12 myoblast differentiation.

The expression of lncRNAs may be governed by transcription factor (TF) binding and promoter‐associated epigenetic mark H3K4me3, or may be mediated by the enhancer‐associated histone modification marker H3K4me1.^33^ Indeed, we observed that changes in DE lncRNA expression were associated with changes in histone modification and TF binding (Figure 2C) through analysis of published ChIP‐seq for TFs (MyoD, MyoG, Cebpb, Usf1 and Max^23^) and histone modifications (H3K4me3^23^ and H3K4me1^24^).

To explore the different mechanisms by which lncRNA expression is regulated, we divided the DE lncRNAs associated with open chromatin into three categories based on the histone ChIP‐seq signal in the open chromatin regions around their TSSs: associated with ATAC and H3K4me1; associated with ATAC and H3K4me3; or associated only with ATAC (Figure 2C). The sum of the first two categories accounted for 82.93% of all open chromatin‐associated DE lncRNAs. The log_2_FC of H3K4me1‐ and H3K4me3‐associated up‐regulated DE lncRNAs on open chromatin were significantly higher than that of down‐regulated ones (Figure 2D). Moreover, the majority of these two categories of DE lncRNAs were correlated with TF binding (MyoD, MyoG, Cebpb, Usf1 or Max), among which over 75% were MyoD/MyoG (Figure 2E). The majority (over 80%) of the MyoD and MyoG binding sites around these two categories of DE lncRNAs were co‐localized in myotubes (Figure 2A,F, top), and their binding motifs were significantly enriched (Figure 2F, bottom). In light of these findings, we concluded that MyoD binding and MyoG binding were critical for the differential expression of lncRNAs.

For the third category of DE lncRNAs, that is, associated with ATAC only, the difference between up‐ and down‐regulated DE lncRNAs in the ATAC‐seq signal was non‐significant, although the distribution was similar to that of the H3K4me1‐ and H3K4me3‐associated categories (Figure 2C). However, the TF binding results for this category were completely different in that the proportion of DE lncRNAs associated with MyoD or MyoG binding was substantially lower than the other two categories. In addition, recognition motifs of other TFs were significantly enriched in these ATAC‐seq peak regions (Figure 2G), and the expression levels of these TFs changed over the course of C2C12 myoblast differentiation (Figure 2H).

### 3.3 The correlations between DE lncRNAs and genes were revealed by WGCNA and Pearson's analysis

**FIGURE 3:**
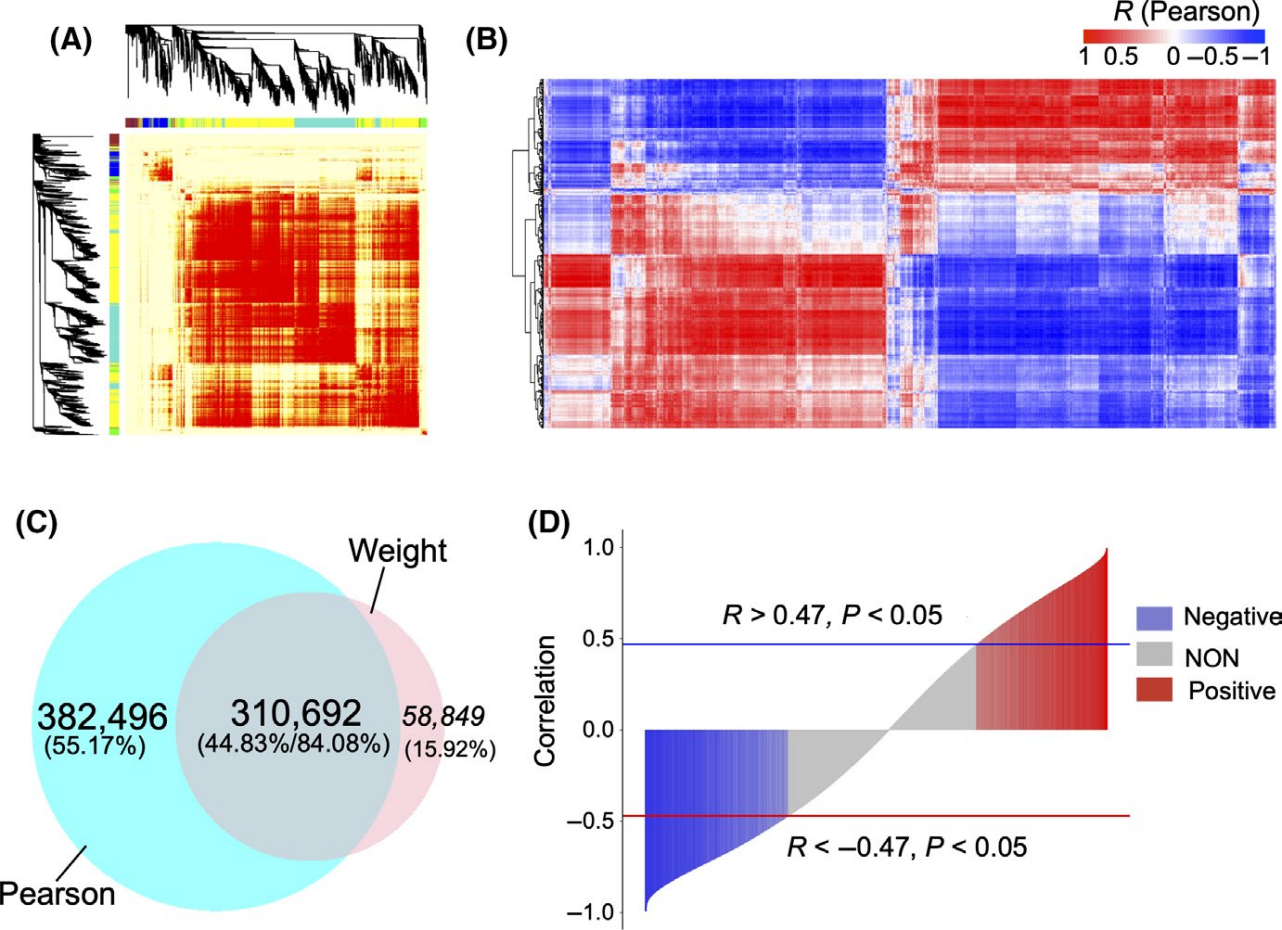
Correlation analysis of differentially expressed (DE) lncRNAs and protein‐coding genes identified over a time course spanning myogenic differentiation in C2C12 myoblasts. A, WGCNA analysis showing the correlation between DE lncRNAs and protein‐coding genes. High correlation is indicated in red. B, Pearson's correlation between DE lncRNAs and protein‐coding genes. Rows and columns represent the DE lncRNAs and protein‐coding genes, respectively. Red and blue indicate positive and negative correlations, respectively. C, Venn diagram showing the overlap between WGCNA and Pearson's analysis. D, Plot of positive and negative correlations of DE lncRNA and gene pairs from both WGCNA and Pearson's analyses. Blue and red bar plots indicate negatively and positively correlated pairs, respectively

To explore the relationship between the expression of lncRNAs and protein‐coding genes during myogenic differentiation, we conducted weighted correlation network analysis (WGCNA) and Pearson's correlation analysis on 385 DE lncRNAs and 3042 DE protein‐coding genes. The WGCNA divided the relevant lncRNAs and protein‐coding genes into five modules consisting of 369 541 highly correlated DE lncRNA‐gene pairs (Figure 3A). The Pearson correlation analysis revealed a significant correlation for 693 188 DE lncRNA‐gene pairs at the transcriptional level (*P* < .05; Figure 3B). Moreover, nearly 84% of the correlated DE lncRNA‐gene pairs identified by WGCNA were also identified by the Pearson correlation analysis (Figure 3C), including both positively and negatively correlated DE lncRNA‐gene pairs from the Pearson analysis (Figure 3D). These results indicated that WGCNA could be validated by Pearson's correlation analysis. Moreover, WGCNA can explore both direct and indirect correlation between elements, whereas Pearson's correlation tests for direct correlation in transcriptional expression.

### 3.4 Four critical TFs and 149 correlated lncRNAs were identified during C2C12 myoblast differentiation

**FIGURE 4:**
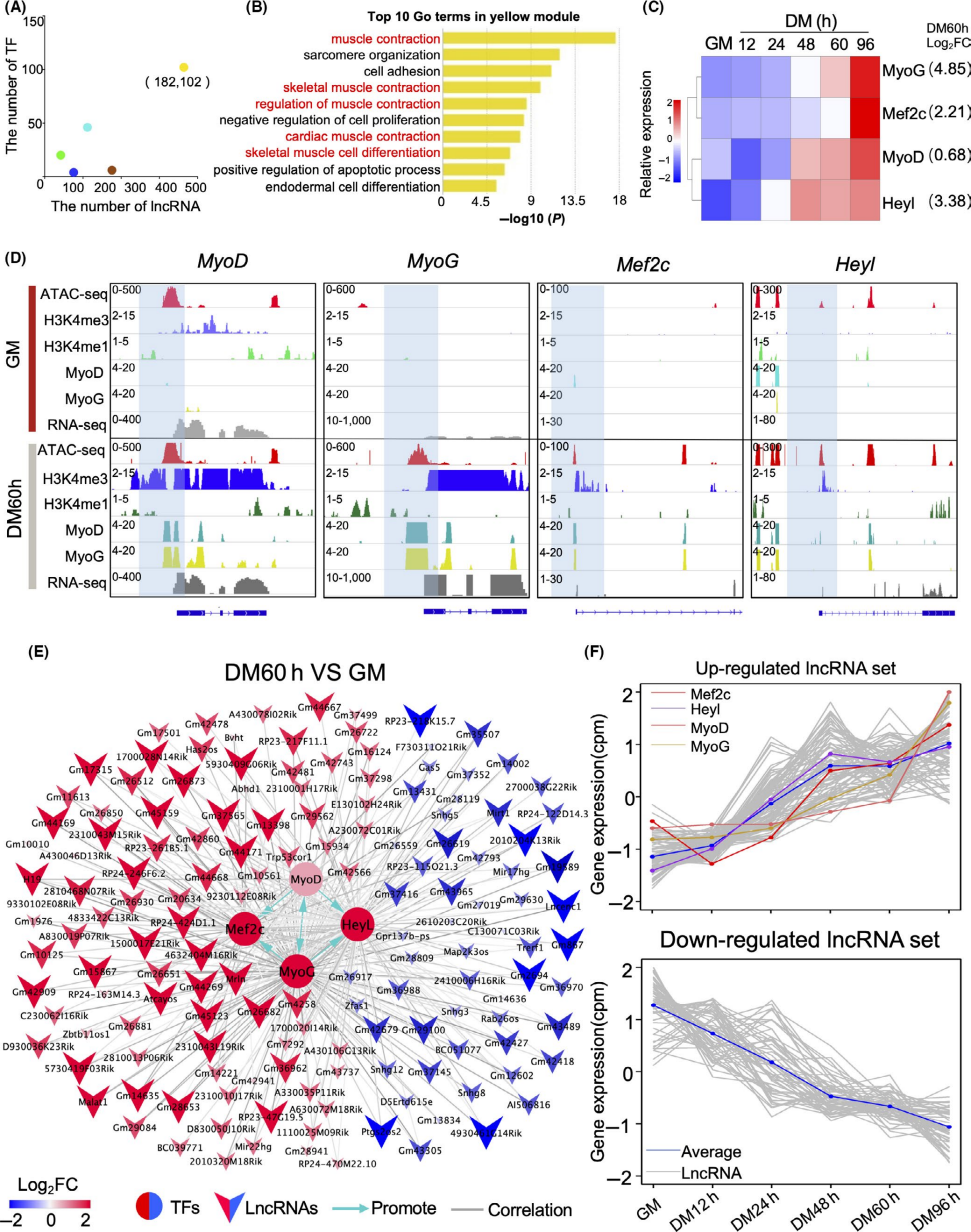
Identification of transcription factors related to myoblast cell differentiation and their correlated DE lncRNAs. A, Scatter plot showing the number of TFs and lncRNAs in each module of WGCNA; the colour of the dots corresponds to the module in WGGCNA. B, Top 10 significant (*P* < .01) GO terms identified among the differentially expressed genes in the yellow module from WGCNA analysis. C, Heat map showing the relative expression profiles of four TFs during myogenic differentiation. The values in parentheses are the log_2_FC of expression levels between proliferation (GM) and differentiation at 60 h (DM60 h). D, Genomic snapshot of the changes in chromatin states around the transcriptional start site (TSS) of *MyoD*, *MyoG*, *Mef2c* and *Heyl* between GM and DM60 h. E, Correlation network showing the changes in expression profiles of TFs and lncRNAs between proliferation (GM) and differentiation 60 h (DM60 h) in C2C12 cells (weight > 0.05, *P* < .01). The red and blue nodes represent the up‐regulated and down‐regulated TF/lncRNAs, respectively. F, Expression patterns of TFs and their correlated DE lncRNAs in (E). The blue line represents the average expression level of DE lncRNAs, while the orange, purple, red and yellow lines represent the relative expression levels of *Mef2c*, *Heyl*, *MyoD* and *MyoG*, respectively

Transcription factors play a crucial role in cell proliferation and differentiation, and the activity of specific TFs can serve as informative markers of cell identity. We calculated the number of DE lncRNAs and TFs in each WGCNA module and found that large numbers of them both appeared in the yellow module (Figure 4A). Moreover, the 10 most significantly enriched GO terms for yellow module involved DE genes that were primarily related to muscle development processes (*P* < .05), including muscle contraction, skeletal muscle contraction and others (Figure 4B). The same analysis of DE genes for other modules revealed radically different results, including no muscle development‐related GO terms among the most significantly enriched terms (Figure 4A, Figure S2). These results strongly suggested that the yellow module was most informative for DE lncRNAs and TFs involved in C2C12 myoblast differentiation.

*MyoD*, *MyoG*, *Mef2c* and *Heyl* were the four TFs involved in skeletal muscle development‐related GO terms identified in the yellow module. These TFs are previously established, significantly up‐regulated markers of myogenic differentiation.^34^, ^35^, ^36^ In the current study, expression of these four TFs was significantly up‐regulated during myoblast cell differentiation, in agreement with previous studies (Figure 4C). Moreover, we found that changes in MyoD and MyoG binding contributed to changes in transcription of *Mef2c*, *Heyl* and themselves (Figure 4D). Change in MyoG expression was also associated with changes in ATAC‐seq and H3K4me3 intensities from the proliferation (GM) to the differentiation (60 hours) stage (Figure 4D).

We found that MyoD and MyoG are crucial TFs for myogenic differentiation, especially through regulation of *Mef2c* and *Heyl* (Figure 4D), which were also previously shown to provide key contributions to myogenic differentiation.^36^, ^37^, ^38^ We thus developed a correlation network between these four TFs and 149 of their correlated DE lncRNAs to improve our understanding of the function of lncRNAs (Figure 4E, Figure S3). Based on the changes in expression between proliferation and differentiation observed in this network, we divided the lncRNAs into an up‐regulated group and a down‐regulated group (Figure 4F).

### 3.5 LncRNAs are involved in the regulation of differentiation of satellite cell during skeletal muscle development

**FIGURE 5:**
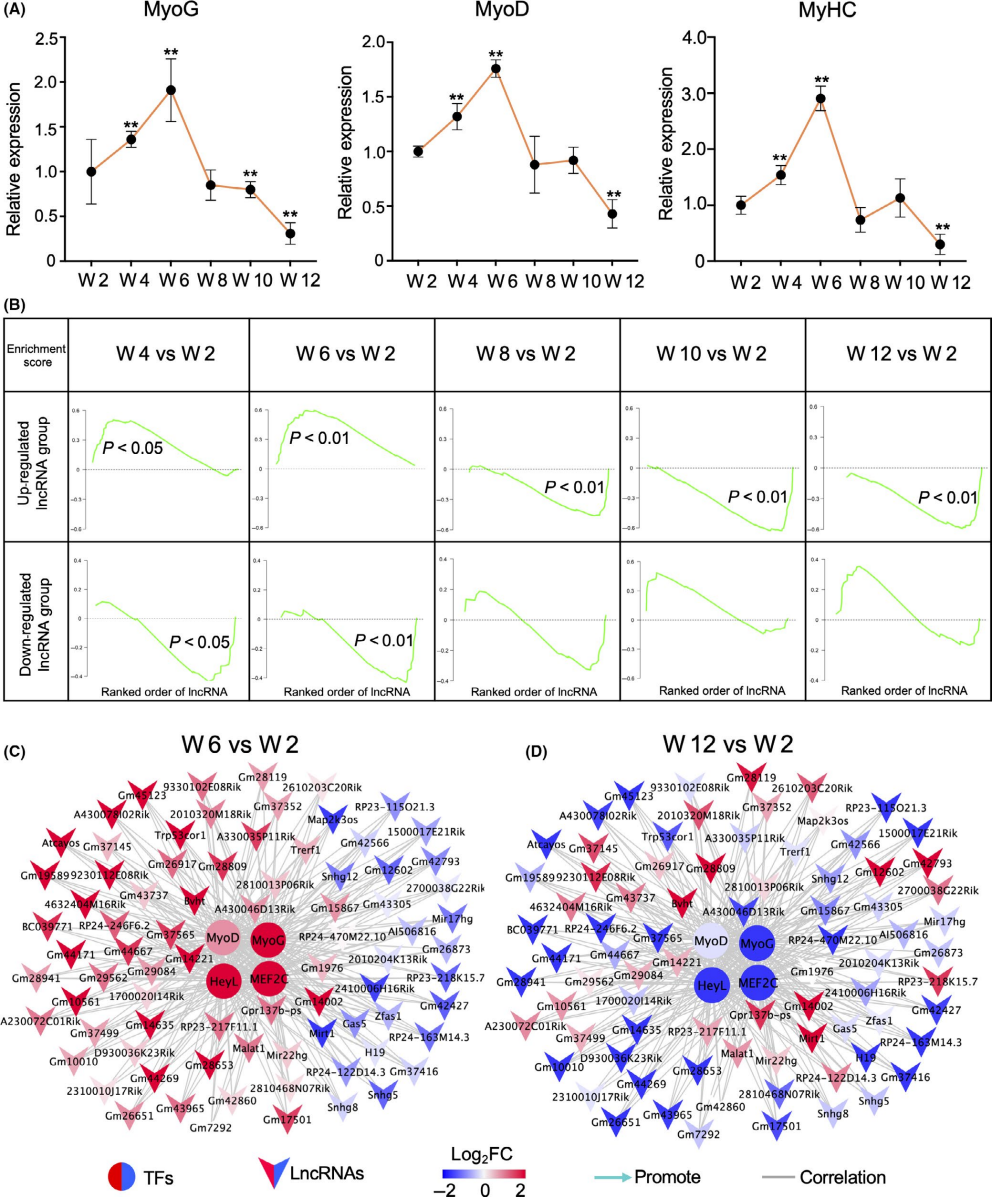
Changes in expression of transcription factors (TFs) and lncRNAs from the TF‐lncRNAs network in satellite cells. A, Expression of *MyoG*, *MyoD* and *MyHC* determined via qPCR in satellite cells during different stages. All comparisons were made with expression levels at 2 wk. Three replicates were used. *Tubulin* was used as the internal control. ‘**’ indicate *P* < .01. B, Gene set enrichment analysis of up‐ and down‐regulated lncRNA groups identified during C2C12 cell differentiation for different stages of satellite cells, including 4‐, 6‐, 8‐, 10‐, and 12 wk compared to expression at 2 wk. The *y*‐axis indicates the enrichment score and the x‐axis indicates rank order of lncRNA. C, D, Network of correlated pairs of TFs and lncRNAs in satellite cells at 6 (C) and 12 wk (D) compared with those at 2 wk

Satellite cells are essential components of skeletal muscle development and regeneration. To investigate the functional differences of satellite cells at different ages in mice, we followed the instruction from a previous study to isolate quiescent satellite cells from mouse skeletal muscle and adherent culture to obtain activated satellite cells.^3^ Through expression analysis, we found that *MyoG*, *MyoD* and *MyHC* were all up‐regulated in the satellite cells of 4‐ and 6‐week‐old mice compared with those in 2‐week‐old mice, indicating the rapid growth of skeletal muscle tissues (Figure 5A). Thereafter, they were down‐regulated, commensurate with age‐related decreases in proliferation and the capacity for satellite cell differentiation. These results were consistent with our previous results generated from satellite cells of the same developmental stage which showed the capability of mouse muscle satellite cells to self‐renew, as well as a significant decrease in their ability to differentiate after a rapid period of growth (4‐6 weeks).^3^

We further investigated whether or not the lncRNAs involved in our TF‐lncRNA correlation network constructed with C2C12 differentiation data were involved in the regulation of the mouse satellite cell differentiation capacity. Our results showed that the up‐regulation and down‐regulation of the lncRNAs in the TF‐lncRNA network were significantly concordant (*P* < .05) between the satellite cells in different rapid growth stages (4‐6 weeks) and differentiated C2C12 via gene set enrichment analysis (Figure 5B,C, Figure S4A). Moreover, these lncRNA‐correlated TFs exhibited the same changes as the lncRNAs (Figure 5C,D, Figure S4). This finding thus indicated that these TFs had a regulatory function in the proliferation and differentiation of myogenic cells.

In the satellite cells from 8 to 12 week mice, the up‐ and down‐regulation of TFs and lncRNAs identified in this correlation network were opposite to those in the satellite cells from 2‐week‐old mice (Figure 5B,D, Figure S4B,C), and especially so for lncRNAs which were up‐regulated in the rapid growth stages of satellite cells. These results further suggested that these TFs and lncRNAs were also involved in the regulation of differentiation capacity decrease of satellite cells with age.

### 3.6 Correlation of TF‐lncRNA expression was validated by qPCR in C2C12 and satellite cells

**FIGURE 6:**
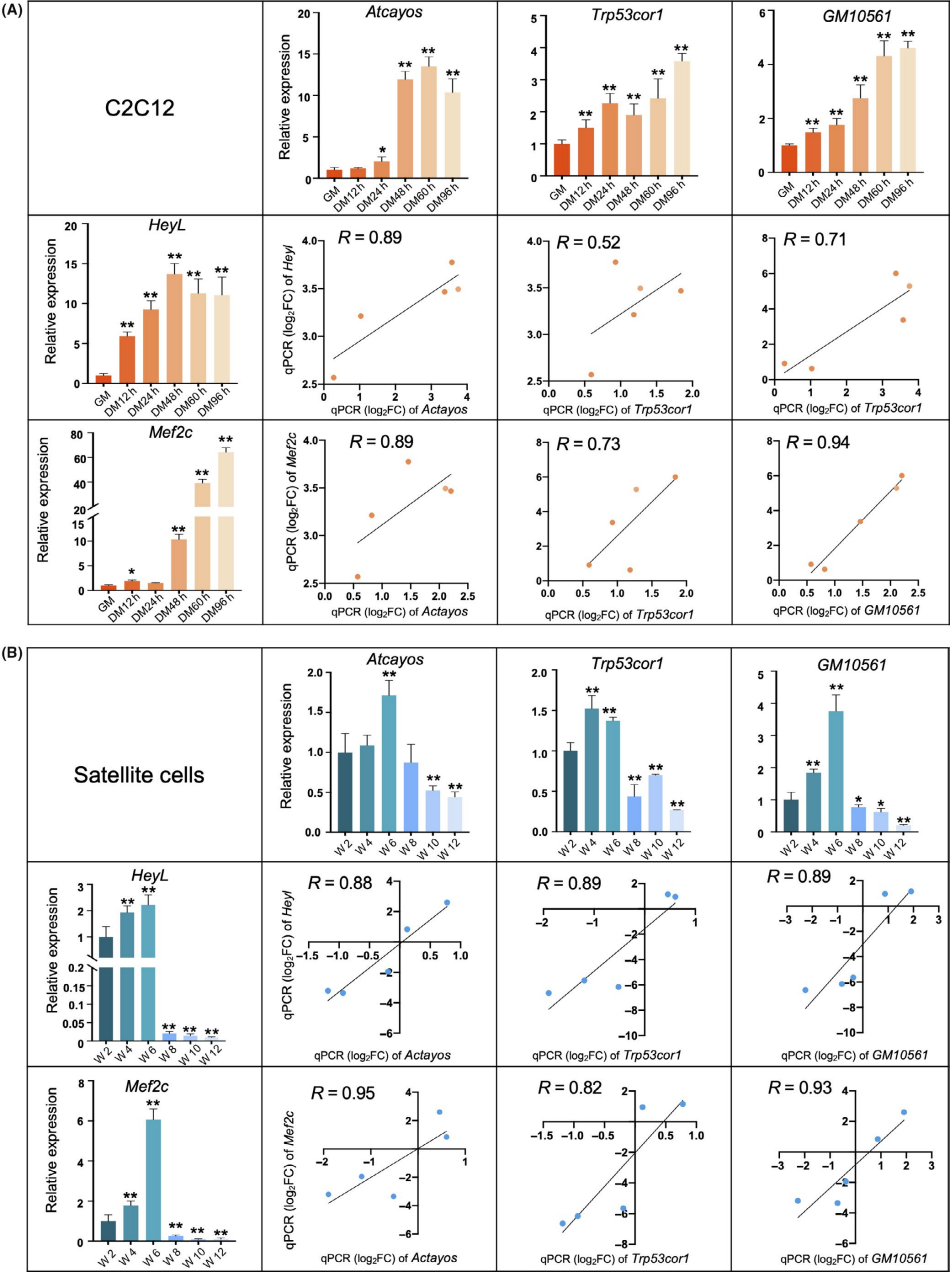
qPCR relative expression analysis of transcription factors (TFs) and lncRNAs in C2C12 myoblasts and satellite cells. Three biological replicates were used for each analysis. *Tubulin* was used as the internal control. The ‘*’ and ‘**’ indicate *P* < .05 and *P* < .01, respectively. Scatter plots show correlations in expression between *Heyl* or *Mef2c* and lncRNAs in C2C12 myoblasts and satellite cells. A, Expression of TFs (*Mef2c* and *Heyl*) and lncRNAs (*Atcayos*, *Trp53cor1*, and *GM10561*) determined via qPCR in the C2C12 cell line. All comparisons were made with expression levels at the proliferation stage (GM). B, The results of qPCR and correlation of relative expression between TFs (*Mef2c* and *Heyl*) and lncRNAs (*Atcayos*, *Trp53cor1*, and *GM10561*) in satellite cells. Fold change is based on comparisons with expression in satellite cells from 2‐week‐old mice

To validate the expression analysis of the four TFs and their correlated C2C12 differentiation‐associated DE lncRNAs, we performed qPCR to measure the expression of *Mef2c* and *Heyl* and the lncRNAs *Atcayos*, *Trp53cor1* and *GM10561* in C2C12 and mouse satellite cells. The expression of those TFs and lncRNAs all changed significantly (*P* < .01) over the course of C2C12 differentiation and across different ages of the mouse satellite cells (Figure 6A,B). Moreover, the expressions of these TFs and lncRNAs, which peaked at week 6, were all positively correlated, in agreement with our data analysis, which also showed a significant correlation between the expression of these TFs and lncRNAs (Figure 6A,B). Together, these results indicated that our expression analysis was reliable.

### 3.7 Inhibition of *Atcayos* and *Trp53cor* reduced the differentiation of satellite cells

**FIGURE 7:**
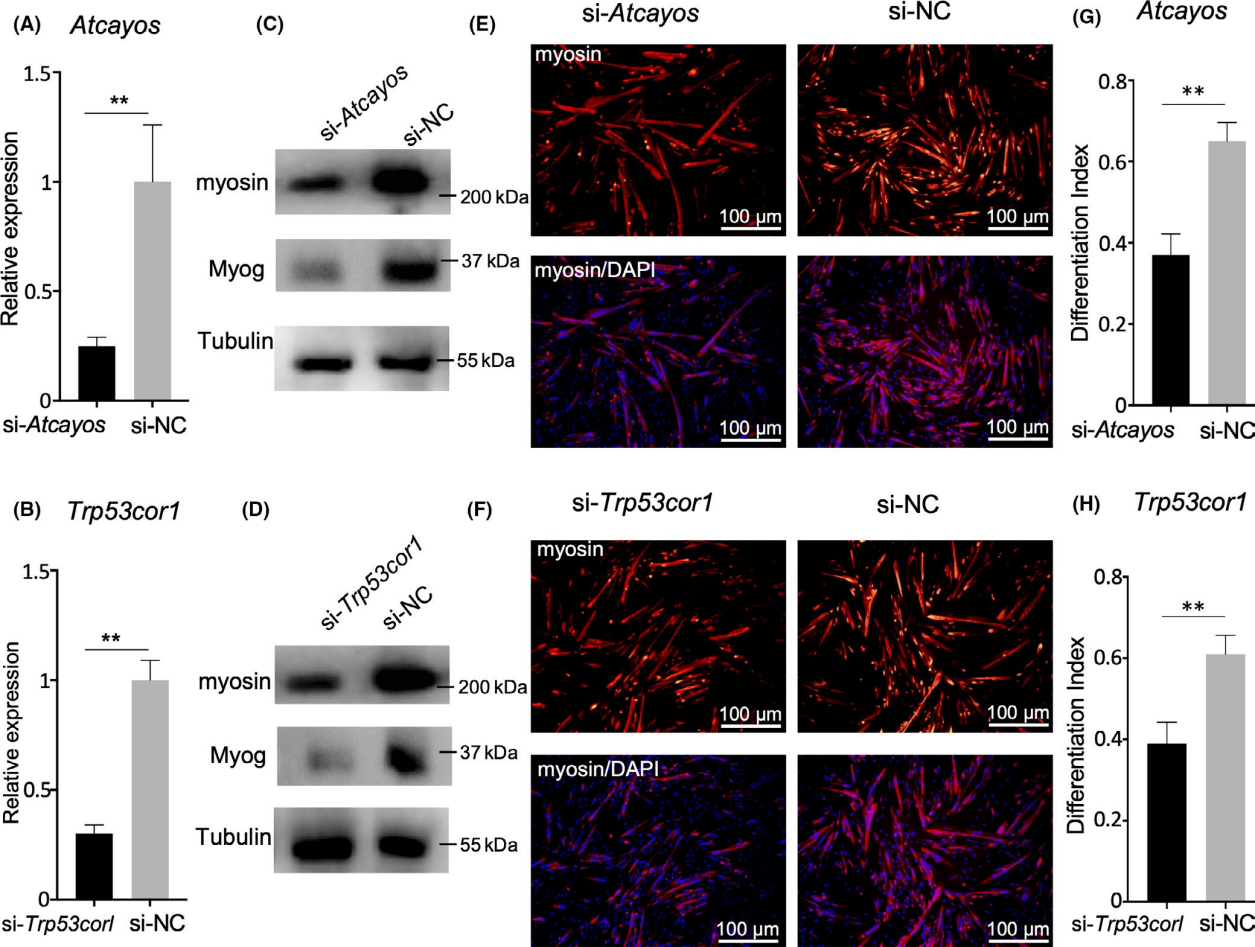
Inhibition of *Atcayos* and *Trp53cor1* in the differentiated satellite cells. A, B, qPCR results of Atcayos and Trp53cor1 expression in differentiated satellite cells at 24 h when *Atcayos* or *Trp53cor1* were inhibited using siRNA, ‘**’ indicate *P* < .01. C, D, Western blots of *Atcayos* and *Trp53cor1* in siRNA knockdown differentiated satellite cells at 24 h. E, F, Immunofluorescence staining of myosin (red) in the differentiated satellite cells at 24 h when *Atcayos* or *Trp53cor1* were inhibited using siRNA. Nuclei were stained with DAPI (blue) Scale bars: 100 μm. Magnification: 100×. G, H, Differentiation index (no. of nuclei in myosin^+^ cell/total nuclei) for satellite cells in (E) and (F)

To further explore the contribution of lncRNAs in the capacity for differentiation among satellite cells, we chose the lncRNAs *Atcayos* and *Trp53cor*, which were up‐regulated in rapid growth stages, for further loss of function assays. The expression of *Atcayos* and *Trp53cor1* was successfully knocked down using RNAi (Figure 7A,B). The Western blot results showed that myosin decreased when *Atcayos* or *Trp53cor1* were inhibited (Figure 7C,D), which was confirmed using immunofluorescence microscopy (Figure 7E‐H). These results showed that inhibition of the TF‐associated lncRNAs *Atcayos* and *Trp53cor1* led to the delayed differentiation of satellite cells, which also indicated that these two lncRNAs participated in positive regulation of the differentiation of satellite cells.

## 4 DISCUSSION

**FIGURE 8:**
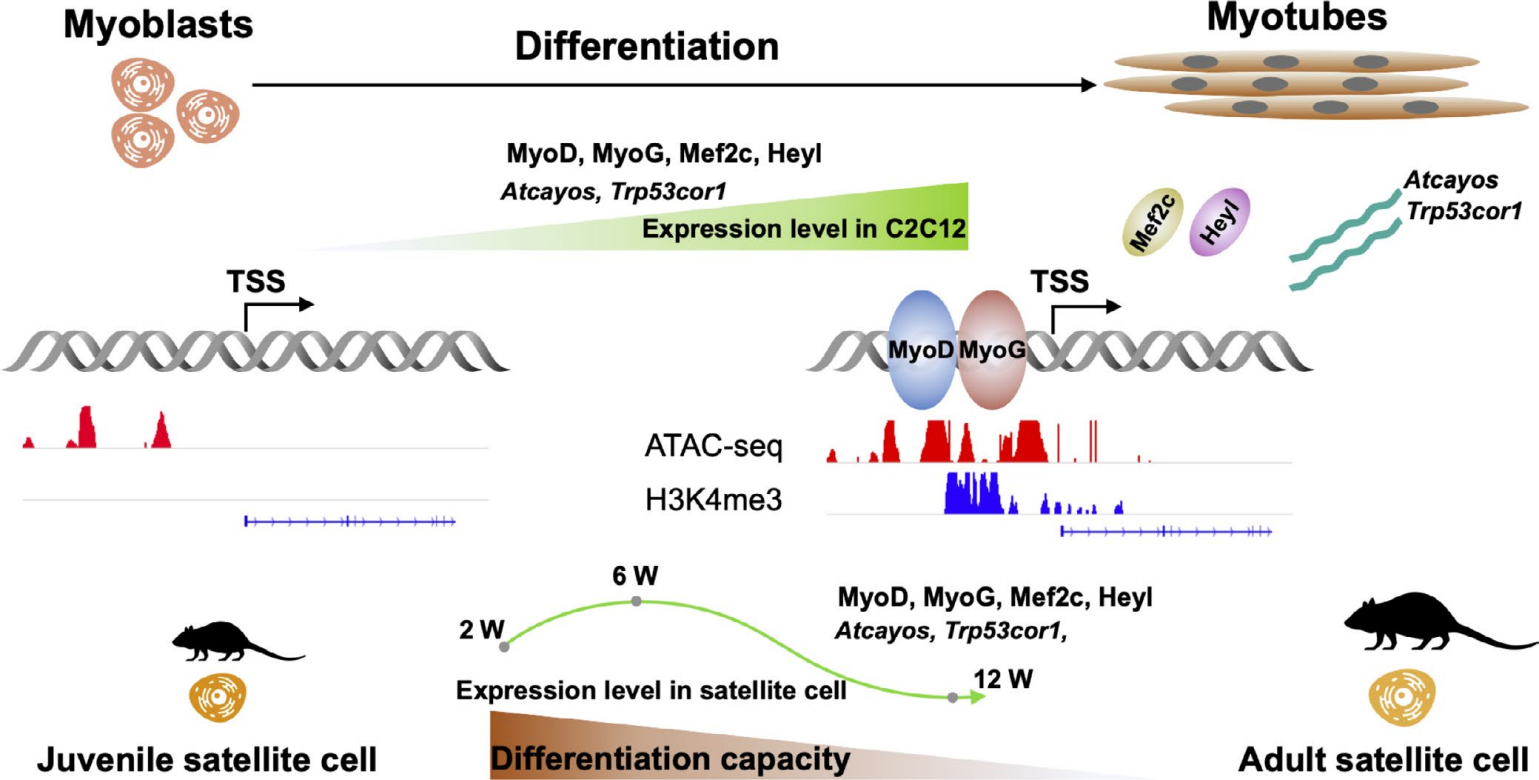
Schematic of TF (MyoD, MyoG, Mef2c, and Heyl) and lncRNA (*Atcayos* and *Trp53cor1*) regulatory interactions in differentiating C2C12 and ageing satellite cells

In the current study, we examined the differential expression patterns of lncRNAs during C2C12 myoblast differentiation to better understand how their transcription is regulated during this developmental stage in mammalian cells. To this end, we combined RNA‐seq to identify DE lncRNAs and TFs during differentiation, with ATAC‐seq to identify open, actively transcribed regions of chromatin, and with ChIP‐seq to determine the histone methylation states in chromatin regions associated with TF binding, which are necessary for differential expression of lncRNAs. We found that the differential expression of lncRNAs during myoblast differentiation was controlled by changes in chromatin states, including the binding of TFs such as MyoD and MyoG. Our analysis further revealed key TFs and their correlated DE lncRNAs associated with myogenic differentiation, which were involved in the regulation of transcription of satellite cells during skeletal muscle development (Figure 8).

Previous studies reported that the expression activation of lncRNA was associated with H3K4me3,^39^ H3K4me1,^40^ etc, histone modifications. In our study, a small fraction of DE lncRNAs (17.07%) related to myoblast differentiation did not associate with H3K4me3 and H3K4me1 but only associated with ATAC signals at their TSS regions. Previous study also reported that lncRNA expression is regulated by TFs in a similar way of protein‐coding genes.^39^, ^41^ Thus, the regulation of TFs becomes particularly important for above only ATAC‐associated DE lncRNAs. Moreover, the motifs of TFs Runx family (Runx1 and Runx2), ATF3, AP‐1 family (Jund and Fosl1) and CTCF were significantly enriched in the ATAC‐seq peak regions at TSS regions of the above DE lncRNAs. Previous studies showed that the TFs Runx1,^42^ ATF3^43^ and CTCF were related to muscle development. CTCF is essential in mediated chromatin loops,^44^ delimiting enhancer‐promoter interaction,^45^ and is implicated in gene activation.^46^ Thus, we speculated that these TFs play critical regulation roles in expression regulation of lncRNAs which were differentially expressed during myoblasts differentiation but only associated with ATAC‐seq signals.

Many TFs can serve as distinct markers of cell states, and their regulatory function is one of considerable long‐term interests among researchers. We constructed a DE TF‐lncRNA correlation network using WGCNA and Pearson correlation's analysis and observed that the TFs *MyoD*, *MyoG*, *Mef2c* and *Heyl* were all significantly correlated with DE lncRNA expression during myoblast differentiation. The TFs MyoD, MyoG and Mef2c were all reported to function in promoting myoblast differentiation.^47^, ^48^, ^49^ *Heyl* also has a positive effect on muscle development.^9^, ^10^ Our TF‐lncRNA network indeed contained previously studied lncRNAs including *H19*,^50^ *Malat1*,^51^ *Mrln* ^52^ and *Snhg8*,^53^ which are reportedly involved in skeletal muscle differentiation. These results indicated that correlation analysis and construction of a TF‐lncRNA network could help us to understand the function of lncRNAs.

Analysis of expression patterns conducted in this study suggested that the DE lncRNAs identified during myoblast differentiation and their correlated TFs participate in the process of decreased differentiation capacity of mouse satellite cells with age. Expression of the lncRNAs, which were up‐regulated during C2C12 differentiation, was also active in mouse satellite cells isolated during the rapid growth stages. Furthermore, the capacity for differentiation among the ageing satellite cells decreased over time compared with the earlier, rapid growth stages.^3^ These lncRNAs associated with rapid growth were down‐regulated with age, strongly suggesting that their decrease in expression with age is related to the concurrently decreasing capacity for differentiation in satellite cells.

Previous research has shown that *Trp53cor1* functions in the regulation of vascular smooth muscle cell proliferation and apoptosis,^17^ and also promotes migration of mesenchymal stem cells.^54^ However, the lncRNA *Atcayos* remains functionally uncharacterized at present. We found, using siRNA knockdowns, that *Trp53cor1* and *Atcayos* were both positively associated with the differentiation of satellite cells during rapid growth, thereby improving our current understanding of the regulatory function of lncRNAs in myogenic differentiation.

In conclusion, by combining ATAC‐seq, ChIP‐seq and RNA‐seq data, we found that dynamic changes in the expression of lncRNAs between the developmental stages of myoblast proliferation and differentiation were closely related to chromatin states. In addition, the *MyoD*, *MyoG*, *Mef2c* and *Heyl* TFs and their correlated lncRNAs, especially *Atcayos* and *Trp53cor1,* were involved in the regulation of myogenic differentiation of satellite cells during skeletal muscle development.

## CONFLICT OF INTEREST

The authors declare that they have no conflict of interest.

## AUTHOR CONTRIBUTIONS

XQ and MH conceived and performed the experiments and explained the data. They also drafted, wrote and revised the manuscript and approved the version to be published. YX, YYX and YL analysed and helped explain the data. They also drafted and revised the manuscript and approved the version to be published. DW, YH, HZ, ZW and WZ assisted in the experiments and helped explain the data. They also drafted and revised the manuscript and approved the version to be published. SZ, XL and YZ designed the study and explained the results. They also revised it critically for important intellectual content and approved the version to be published.

## Supporting information

Supplementary Material

## Data Availability

Data are openly available in a public repository that issues data sets with DOIs: ChIP‐seq data of H3K4me3, MyoD, MyoG, Usf1, Max and Cebpb that support the results of this study are openly available in NCBI at https://doi.org/10.1038/nature13992.^23^ ChIP‐seq data of H3K4me1 that support the findings of this study are openly available in NCBI at https://doi.org/10.1016/j.molcel.2013.07.022.^24^ Satellite cell RNA‐seq data that support the findings of this study are openly available in NCBI at https://doi.org/10.3389/fgene.2019.00220.^3^
