# Supplementary material for: LncRNAs are regulated by chromatin states and affect the skeletal muscle cell differentiation
Source: Cell Prolif. 2020 Aug 7;53(9):e12879. doi: 10.1111/cpr.12879 (PMC7507427; doi:10.1111/cpr.12879)
Supplement: Supplementary file 1 — Supplementary Material [file CPR-53-e12879-s001.docx]

# Supplementary

Supplementary Table 1 ATAC-seq data quality control

| sample | Input reads | Total mapped reads | Mapped reads rate | Narrow peaks |
| --- | --- | --- | --- | --- |
| GM_1 | 175720444 | 174573049 | 99.35% | 236461 |
| GM_2 | 231313166 | 229918420 | 99.40% | 244762 |
| DM60h_1 | 203080896 | 201996878 | 99.47% | 254992 |
| DM60h_2 | 332281702 | 330055233 | 99.33% | 280972 |

Supplementary Table 2 Primers used in RT-qPCR experiments

| Name | Forward primer | Reverse primer |
| --- | --- | --- |
| RP23-115O21 | 5′-CCTGCAACAGTGGTGACACA-3′ | 5′-AACCAGTCCAGATGACTGCAA-3′ |
| Malat1 | 5′-GCAGTGTGCCAATGTTTCGT-3′ | 5′-GCTGTTTCCTGCTCCGAGAT-3′ |
| GM28653 | 5′-GCCATATTTTCGGGGAGCCT-3′ | 5′-TCCTCTGCTTTCGGCTTGTT-3′ |
| GM10125 | 5′-CCACCTCCATGGTGGCTAAG-3′ | 5′-TGCCAGAGAAAACCAGCACT-3′ |
| Mef2c | 5′-CTGATGGGCGGAGATCTGAC-3′ | 5′-TTGCTGCCAGGTGGGATAAG-3′ |
| Heyl | 5′-TGGGCCAAGAGAGCATTACG-3′ | 5′-GCTTCCTCCTGTTTTGCAGAC-3′ |
| Myog | 5’-CAGCAGTTGGTGTGAGGGG-3’ | 5’-CATACAGCTCCATCAGGTCGG-3’ |
| MyoD | 5’-TCCTCATAGCACAGGGGTGA-3’ | 5’-GCAAGCTGTGGGGAAAAGTG-3’ |
| MyHC | 5’-GGACCCACGGTCGAAGTTG-3’ | 5’-CCCGAAAACGGCCATCT-3’ |
| Atcayos | 5′-ACTGAGGGAGACAACAGGGT-3′ | 5′-GCCACTGGGACAATCCATGA-3′ |
| Trp53cor1 | 5′-GGGTGCTCTTGCTGTTCAGT-3′ | 5′-CTGCAGGCTCACTGTCTTGT-3′ |
| GM10561 | 5′-GGCTTTGTCTCACCCAGTTG-3′ | 5′-GCCAGTGACCAGCAAAGATT-3′ |
| β-tubulin | 5′-GACTATGGACTCCGTTCGCTC-3′ | 5′TATTCTTCCCGGATCTTGCTG-3′ |

Supplementary Table 3 Target sequence of si-LncRNA

| Name | Target sequences |
| --- | --- |
| si-Atcayos | GAACCCTCAAGTGCTTAGA\GCTACTACCCAGTCGTTGA\CTGAGTAAGGCTCTCAATA\ TTGAGGTCACTAGGCTACTA\AAGGTCCCAAGAGCACAGAG\AACAAGGGAGACCAGCACAC |
| si-Trp53cor1 | CAAGAACTTGTGGACAACCT\GCCTTGAACCAGTCCCTCCT\GAATGAGTTACTACCTCTGA\ GGCAAGAACTTGTGGACAA\GCGAGAGCATTGACACTTA\GGGTGTTGTTCAGTTGGTA |


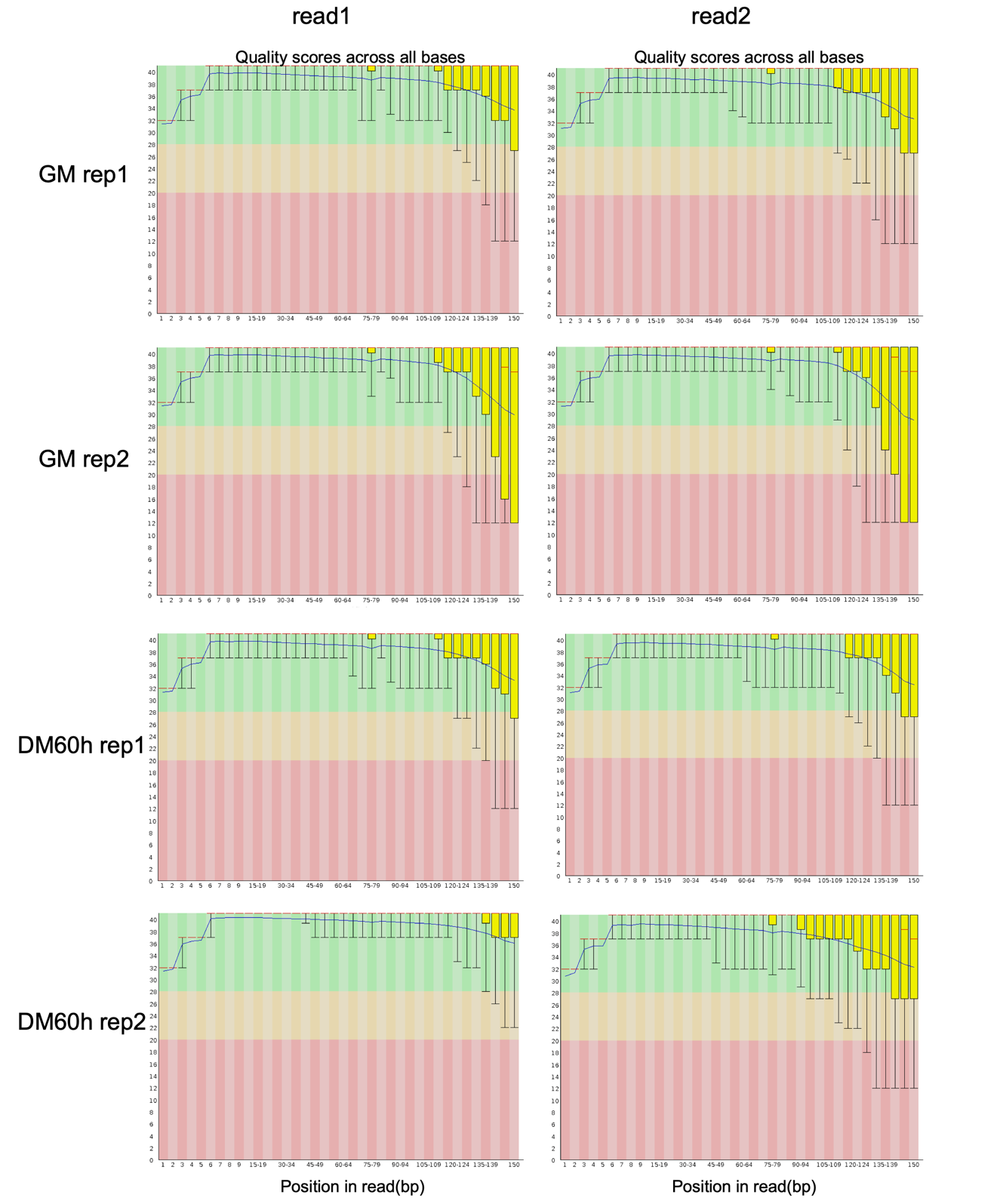


Supplementary Figure 1. The reads quality statistics of ATAC-seq data. The y-axis indicates the sequencing quality value and the x-axis indicates the base position of the reads


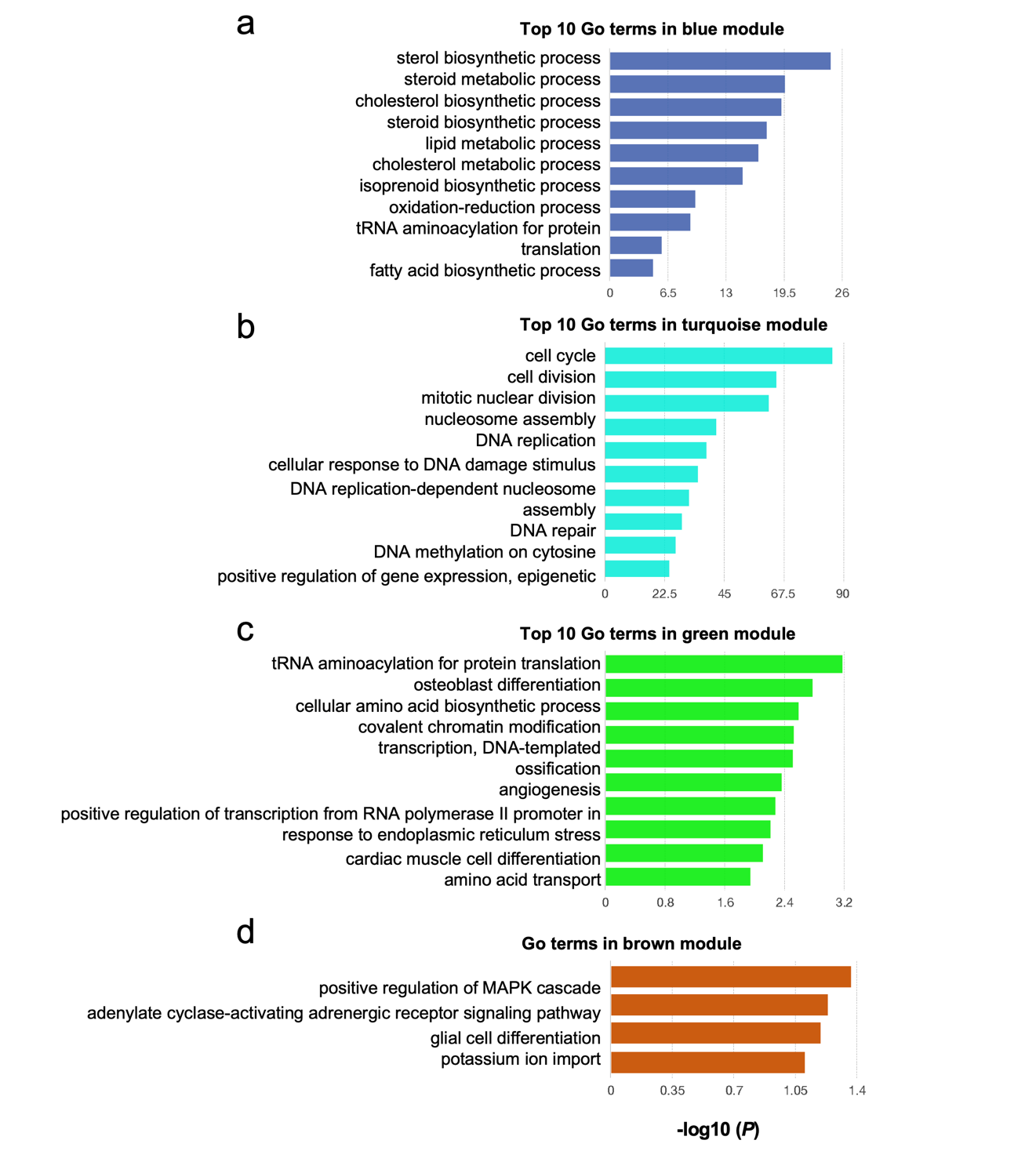


Supplementary Figure 2. (a-d) Top 10 GO terms of the blue module (a), turquoise module (b), green module (c), and all GO terms of the brown module (d) based on WGCNA analysis.


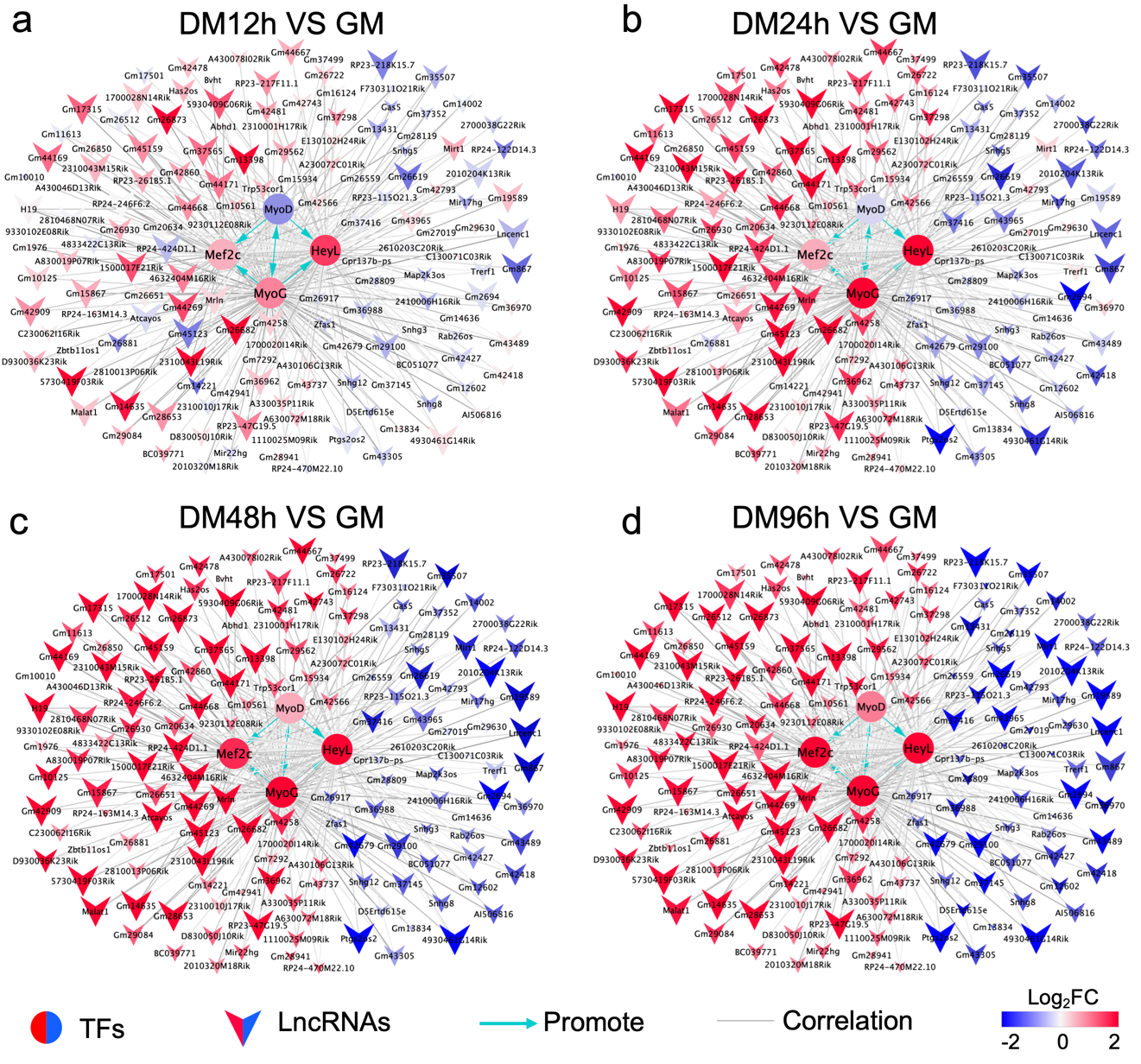


Supplementary Figure 3. TF-lncRNA correlation network in C2C12. (a-d) Correlation network showing the expression changes in TFs and lncRNAs between proliferation and differentiation for 12 (a), 24 (b), 48 (c) and 96 h (d) (weight > 0.05, P < 0.01).


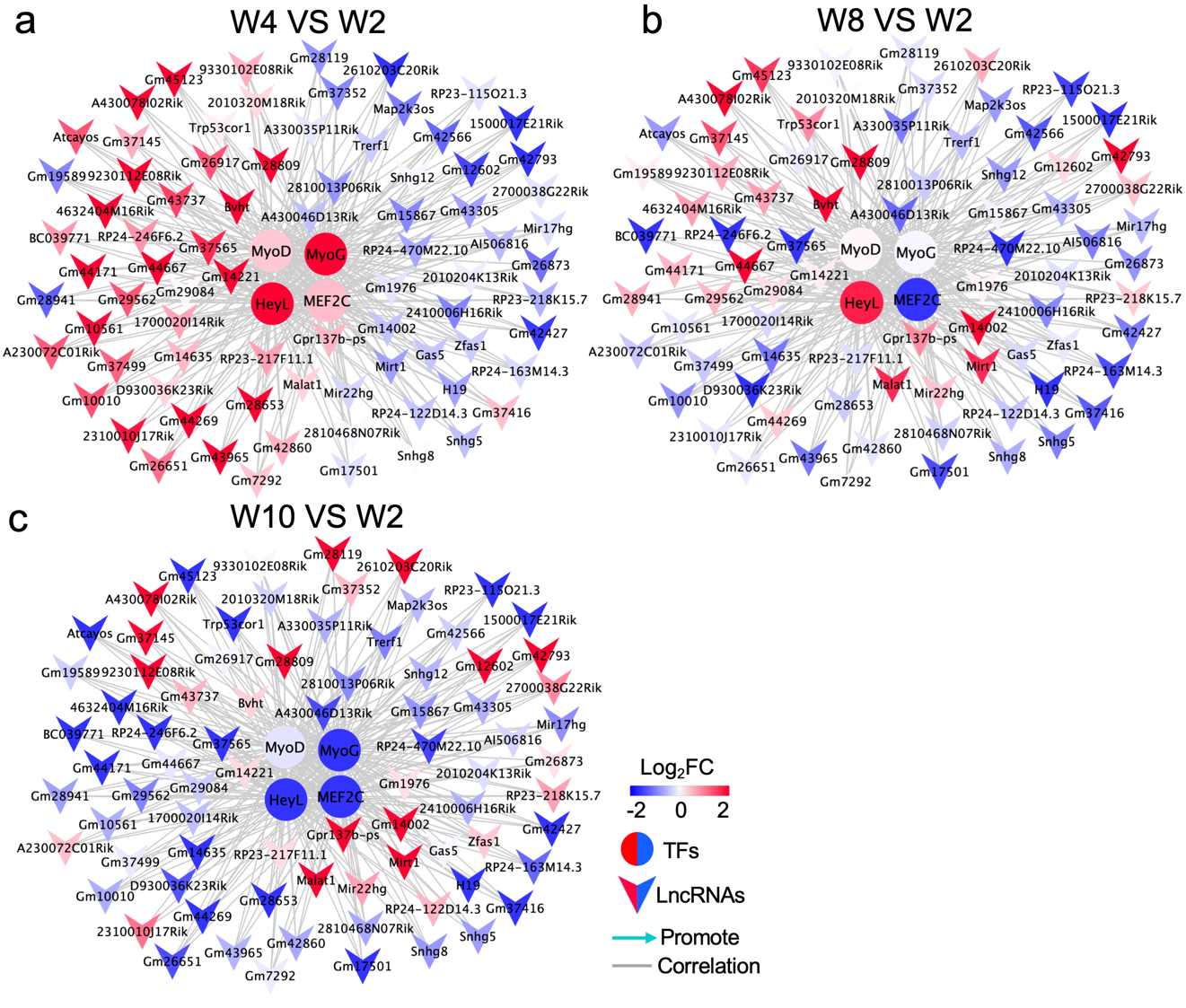


Supplementary Figure 4. Expression changes in the TF-lncRNA correlation network in satellite cells. TFs and lncRNA correlation network in satellite cells at 4 (a), 8 (b) and 10 weeks (c) compared with those at 2 weeks.
